# The Association between Neurocysticercosis and Epilepsy

**DOI:** 10.4269/ajtmh.20-0569

**Published:** 2020-09

**Authors:** Arturo Carpio

**Affiliations:** School of Medicine; University of Cuenca; Cuenca, Ecuador; G. H. Sergievsky Center; Columbia University; New York, New York; E-mail: arturo.carpio@ucuenca.edu.ec

Dear Sir,

I read with interest the review “Unique Characteristics of Epilepsy Development in Neurocysticercosis,” published by Herrick et al.^1^ Neurocysticercosis (NCC) is a neglected zoonotic disease of high public health impact that is a cause of unacceptable morbidity and mortality.^2^ Epilepsy is one of the most common neurological disorders of the brain, and it is also a public health imperative because it carries neurological, cognitive, psychological, and social consequences, and contributes substantially to the world’s burden of disease.^3^ Therefore, information regarding both these diseases should be based on reliable and rigorous scientific data.

According to Herrick et al.,^1^ “NCC is the most common cause of adult-acquired epilepsy in the world.” This statement is not true. The authors based their statement on cross-sectional studies, most of them addressed to determine the prevalence of epilepsy, and some with serious methodological flaws, such as establishment of the diagnosis of NC by serological tests (electroimmunotransfer blot). Diagnosis of NC must be based in neuroimaging, but serological tests may be used for confirmation.^4^ In studying the etiology of epilepsy, it is more appropriate to use incident cases rather than prevalent cases because one cannot distinguish among the potential etiological factors that preceded the onset of epilepsy, and, thus, cause and effect become difficult to establish with certainty. To our knowledge, there are no prospective cohort studies that have measured the risk of seizure recurrence (i.e., epilepsy) in patients with NC.

Consistent with a systematic review addressed to describe the burden of epilepsy, carried out by the “Prevention Task Force of the International League Against Epilepsy,”^5^ stroke was the most common etiology in adults (12%), followed by traumatic brain injury (5.3%) and central nervous system infections (2.4%) in high-income countries. It was also reported that the median proportion of epilepsy cases attributable to NC in lower- and middle-income countries was 34%, based on cross-sectional studies performed in highly endemic communities. Most of these studies used prevalent cases (which is inappropriate, as mentioned earlier) and additionally did not differentiate between acute symptomatic seizures^6^ and recurrent unprovoked seizures (i.e., epilepsy). This distinction is crucial because not all seizures evolve into epilepsy.^6,7^ Indeed, this Task Force warned that the high proportion attributed to NCC should be interpreted with caution, as these studies examined prevalent cases of epilepsy and were limited by low availability of diagnostic tools to detect other causes of epilepsy in the communities studied.^8^

Herrick et al.^1^ affirm that “several studies have demonstrated a benefit of antiparasitic treatment in reducing the frequency of seizures in subjects with NCC.” This is also debatable. A Cochrane review found no significant difference when albendazole was used compared with no treatment for recurrence of seizures in people with viable parenchymal cysts.^9^ Another extensive review on this issue concluded that effectiveness of antihelminthic treatment on seizure outcomes remains uncertain.^10^ A recent prospective study^11^ showed that the association between albendazole treatment and seizure outcomes was nonlinear and changed over time because most cysts either calcify or resolve completely, regardless of whether treated with albendazole. I agree with Herrick et al.,^1^ in the sense that “factors that may also play a role in epilepsy development for patients with NCC include a genetic predisposition toward a lowered seizure threshold…” as we proposed a few years ago.^12^

In conclusion, there is no doubt that seizures are the most frequent symptom of parenchymal NCC and a potential risk factor for epilepsy, which must be confirmed and measured by further prospective cohort studies. Meanwhile, NCC should not be overemphasized as a main cause of epilepsy, which may lead to other possible causes for acquiring epilepsy being ignored and could lead to inappropriate clinical management of people with epilepsy. It is time to conduct a prospective cohort study enrolling patients with new onset seizures and NCC to tell us how many of these patients actually develop epilepsy.
